# Emerging Role for Linear and Circular Spermine Oxidase RNAs in Skeletal Muscle Physiopathology

**DOI:** 10.3390/ijms21218227

**Published:** 2020-11-03

**Authors:** Jonathan Fernando Reinoso-Sánchez, Giulia Baroli, Guglielmo Duranti, Silvia Scaricamazza, Stefania Sabatini, Cristiana Valle, Mariangela Morlando, Robert Anthony Casero, Irene Bozzoni, Paolo Mariottini, Roberta Ceci, Manuela Cervelli

**Affiliations:** 1 Department of Science, “Department of Excellence 2018–2022”, University of Rome “Roma Tre”, 00146 Rome, Italy; jonathanfernando.reinososanchez@uniroma3.it (J.F.R.-S.); giulia.baroli@uniroma3.it (G.B.); paolo.mariottini@uniroma3.it (P.M.); 2 Laboratory of Biochemistry and Molecular Biology—Department of Movement, Human and Health Sciences, University of Rome “Foro Italico”, 00135 Rome, Italy; guglielmo.duranti@uniroma4.it (G.D.); stefania.sabatini@uniroma4.it (S.S.); roberta.ceci@uniroma4.it (R.C.); 3 Department of Biology, University of Rome “Tor Vergata”, 00133 Rome, Italy; silviascaricamazza@gmail.com; 4 IRCCS Fondazione Santa Lucia, 00179 Rome, Italy; c.valle@hsantalucia.it; 5 National Research Council, Institute of Translational Pharmacology (IFT), 00133 Rome, Italy; 6 Department of Pharmaceutical Sciences, “Department of Excellence 2018–2022”, University of Perugia, 06123 Perugia, Italy; mariangela.morlando@unipg.it; 7 Sidney Kimmel Comprehensive Cancer Center, Johns Hopkins University, Baltimore, MD 21287, USA; rcasero@jhmi.edu; 8 Department of Biology and Biotechnology “Charles Darwin”, University of Rome “La Sapienza”, 00185 Rome, Italy; irene.bozzoni@uniroma1.it; 9 Center for Life Nano Science@Sapienza, Istituto Italiano di Tecnologia, 00161 Rome, Italy

**Keywords:** circRNA, spermine oxidase, skeletal muscle atrophy, amyotrophic lateral sclerosis murine models

## Abstract

Skeletal muscle atrophy is a pathological condition so far without effective treatment and poorly understood at a molecular level. Emerging evidence suggest a key role for circular RNAs (circRNA) during myogenesis and their deregulation has been reported to be associated with muscle diseases. Spermine oxidase (SMOX), a polyamine catabolic enzyme plays a critical role in muscle differentiation and the existence of a circRNA arising from *SMOX* gene has been recently identified. In this study, we evaluated the expression profile of circular and linear SMOX in both C2C12 differentiation and dexamethasone-induced myotubes atrophy. To validate our findings in vivo their expression levels were also tested in two murine models of amyotrophic lateral sclerosis: SOD1^G93A^ and hFUS^+/+^, characterized by progressive muscle atrophy. During C2C12 differentiation, linear and circular SMOX show the same trend of expression. Interestingly, in atrophy circSMOX levels significantly increased compared to the physiological state, in both in vitro and in vivo models. Our study demonstrates that SMOX represents a new player in muscle physiopathology and provides a scientific basis for further investigation on circSMOX RNA as a possible new therapeutic target for the treatment of muscle atrophy.

## 1. Introduction

The development of skeletal muscle (SM) fibers is a multistep process that requires the fusion of myoblasts to produce multinucleated muscle fibers with contractile properties. Numerous signaling factors act at various points during muscle differentiation by regulating proliferation, muscle-specific differentiation genes, and sarcomeric activation to finally establish the SM phenotype [1,2]. Among these factors, our previous study showed that spermine oxidase (SMOX, EC number 1.5.3.16) plays a critical role in SM differentiation [3]. SMOX is an enzyme that specifically oxidizes the natural substrate spermine with the production of spermidine, hydrogen peroxide and the aldehyde 3-aminopropanal [4,5] and therefore plays a crucial role in the homeostasis of polyamines in mammals [6,7]. It has been reported that SMOX activity maintains basal SM gene expression; indeed a reduction in SMOX is sufficient to induce muscle fiber atrophy [8], that is the wasting or loss of muscle mass resulting in muscle weakness and general muscle fatigue [9,10]. Interestingly, the cyclin-dependent kinase inhibitor p21, a protein highly induced during muscle atrophy, negatively regulates SMOX expression [2,8]. By contrast, forced expression of SMOX increases muscle fiber size in multiple models of muscle atrophy. 

Recently it has been noted the involvement and importance of non-coding RNAs (ncRNAs) as regulators of myogenesis [11,12,13,14]. Many studies have focused on a particular type of ncRNAs, namely, circular RNAs (circRNAs); they are produced by a non-canonical splicing event also known as back-splicing, in which a joining between a downstream splice donor and an upstream acceptor site occurs [15,16]. Because of their covalently closed structure, circRNAs are highly stable as indicated by their peculiar resistance to exonuclease activities. Different regulatory functions have been ascribed to circRNAs both in the nucleus and in cytoplasm: they can control the transcription and processing of their host transcripts, they can act as sponges/scaffolds for microRNAs (miRNAs) and proteins, and finally circRNAs can be also translated even though still few cases are known so far [17]. Remarkably, several studies showed that circRNAs are highly present in skeletal muscle; their expression changes during myogenesis [18,19] and their abnormal expression play an important role in the muscle atrophy process [20]. Notably, in two recent studies regarding muscle atrophy and transient focal ischemia in murine models [21,22], the existence of a circRNA arising from SMOX gene has been revealed. These findings led us to investigate the expression of linear and circular SMOX and their ratio during myogenesis and atrophy to contribute to unveiling the mechanism underlying the two processes. The molecular mechanism of atrophy can be studied in vitro by treating muscle cells with the synthetic glucocorticoid dexamethasone (DEXA), a well-known anti-inflammatory drug that promotes the degradation of proteins related to muscle mass [23,24] via the ubiquitin-proteasome proteolytic pathway. Particularly, two muscle ubiquitin ligases have been associated with muscle atrophy, namely, Atrogin1 and muscle RING-finger protein-1 (MuRF-1); their activation through DEXA leads to a reduction in muscle mass [25,26,27]. Thus, expression levels of these two factors allows researchers monitoring the effectiveness of the DEXA treatment. 

DEXA-treated mouse C2C12 myotubes have been largely used as a model system to investigate mechanisms of muscle wasting [26,28]. Indeed, the metabolic changes induced by DEXA in these cells are similar to those observed in atrophying muscle in animal models and human pathological conditions [29,30,31,32]. Among these latter, Amyotrophic Lateral Sclerosis (ALS) is an adult neurodegenerative disease, ultimately leading to death approximately three years after onset [33,34]. Notably, progressive muscle atrophy is an early symptom in ALS. This disease is currently incurable despite intense research and numerous but unsuccessful clinical trials.

Here, we hypothesized that the expression of linear SMOX and circular SMOX could be regulated, in opposite way during myogenesis and atrophy process, by compensatory mechanisms resulting in increased linear SMOX expression when circular SMOX is suppressed and vice versa.

Hence, the aim of the present study was to evaluate the presence of a circular RNA derived from SMOX gene and its involvement as a potential regulator of muscle during differentiation and in atrophic conditions in in vitro and in vivo models, also deepening the relation between SMOX and p21 expression in muscle. Circular and linear SMOX and p21 transcript analyses has been evaluated in C2C12 cells during in vitro muscle differentiation and in atrophic condition upon DEXA treatment. Moreover, the expression of these genes was evaluated in vivo employing two murine models of ALS, the *SOD1^G93A^* and the *hFUS^+/+^*, as they recapitulate most of the ALS neurodegenerative processes found in patients [35,36].

## 2. Materials and Methods

### 2.1. Materials

All chemical reagents, unless specified otherwise, were purchased from Sigma-Aldrich Chemical (Sigma-Aldrich, St. Louis, MO, USA).

### 2.2. Plasmid Constructions

The p-cSmox construct was generated by cloning the Exons 2 and 3 of *SMOX* gene into pCR 2.1 vector (TA Cloning Kit, Invitrogen, Carlsbad, CA, USA) using the primers circSMOX3 and circSMOX4 listed in Table 1. The plasmid was amplified in DH5α competent cells (Thermo Fisher Scientific, Waltham, MA USA). Digestion analysis was performed with *Hin*dIII and *Xho*I on the cloned product in pCR2.1 (p-cSMOX) to confirm the cloning and then double-strand sequenced by the European Division of Macrogen Inc. (Amsterdam, The Nederland).

### 2.3. Cell Culture

C2C12 myoblasts (ATCC, Manassas, VA, USA) were cultured in 25 cm^2^ culture flasks with growth medium: Dulbecco’s-modified Eagle’s medium (DMEM; HyClone—Cytiva Europe GmbH, Buccinasco (MI), Italy) supplemented with Glutamax-I (4 mM l-alanyl-l-glutamine), 4.5 g/L glucose (Invitrogen, Carlsbad, CA, USA) and 10% heat-inactivated fetal bovine serum (FBS; HyClone-Cytiva Europe GmbH, Buccinasco (MI), Italy). The cells were incubated at 37 °C with 5% CO_2_ in a humidified atmosphere. Cells were split 1:6 twice weekly and fed 24 h before each experiment. Differentiation into myotubes was achieved by culturing pre-confluent cells (85% confluency) in differentiation medium containing 2% FBS and monitoring them by microscopy and for myosin expression [37]. To induce myotubes atrophy, after 96 h in differentiation medium, C2C12 myotubes were treated with DEXA (DM-D) or vehicle (DM-C) for the following 24 h. Briefly, DEXA was dissolved in methanol at a stock concentration of 50 mM immediately before use and then diluted in the culture medium to a final concentration of 100 μM. At this working solution, neither methanol concentration (0.1%, v/v) nor DEXA show any toxic effect on myotubes. Samples for protein and RNA analysis were obtained from C2C12 myotubes at different stage of differentiation (from 6 to 168 h in differentiation medium) and after DEXA/methanol exposure. 

### 2.4. Cell Protein Extraction 

Cells were washed twice with ice-cold PBS and then lysed in RIPA buffer (150 mM NaCl, 50 mM tris-HCl pH 8, 1 mM EDTA, 1% NP40, 0.25% sodium deoxycholate, 0.1% SDS, water to volume), supplemented with protease and phosphatase inhibitor cocktails in ice. The resulting lysates were centrifuged at 14,000× *g* for 10 min at 4 °C, and then utilized for protein analysis. An aliquot of cell lysates was tested for protein content using the Bradford method using bovine serum albumin as the standard (Sigma-Aldrich, St. Louis, MO, USA).

### 2.5. ALS Mouse Models 

All animal procedures were performed accordingly to the European Guidelines for the use of animals in research (2010/63/EU) and to the requirements of Italian laws (D.L. 26/2014). The ethical procedures were approved by the office of the Animal Welfare, the Department of Public Health and Veterinary, the Department of General Management of Animal Care and Veterinary Drugs of the Italian Ministry of Health (protocol number 931/2017/PR). Mice were housed in our indoor virus/antigen-free animal facility at constant temperature (22  ±  1 °C) and relative humidity (50%) with 12-h light cycle (light 7 a.m.–7 p.m.). Food and water were provided ad libitum. Adult transgenic mice expressing human mutated SOD1^G93A^ (B6.Cg-Tg(SOD1 G93A)1Gur/J) were obtained from The Jackson Laboratory (Bar Harbor, ME, USA) and bred in our animal facility. For the generation of experimental SOD1^G93A^ animals, hemizygous transgenic males were crossbred with C57BL/6 females and progeny was genotyped by PCR [38]. Hanging grid test, starting at 55 day of age, was performed to evaluate disease onset [39]. In order to follow disease progression, behavioral scores and body weight were monitored according to Apolloni et al. [40]. In our experimental disease assessment, mice showed onset at 91.7 ± 2.9 days, early symptomatic stage at 121.1 ± 1.7 days, symptomatic stage at 145.4 ± 2.1 days and survival/end stage at 168.7 ± 1.9 days. Adult mice expressing hemagglutinin-tagged human wild-type FUS (Tg (Prnp-FUS) WT3Cshw/J) [36] were obtained from The Jackson Laboratory (Bar Harbor, ME, USA) and genotyped as previously described [41]. Mice were maintained in hemizygosity on the genetic background C57BL/6 and were backcrossed to obtain homozygous mice, utilized as experimental animals. In our housing conditions, hFUS mice have the onset at 35 ± 4 days and a survival of 40.2 ± 5.8 days according to [41]. In order to overcome any possible gender-dependent mixed results, we used only male mice with both strains. Mice were anaesthetized and then sacrificed for tissue dissection; gastrocnemius muscle was dissected and immediately frozen in liquid nitrogen.

### 2.6. RNA Isolation, Reverse Transcription and qReal-Time PCR

Total RNA from C2C12 cells and gastrocnemius muscle from *SOD1^G93A^* and *hFUS^+/+^* was extracted using TRIzol Reagent (Invitrogen, Carlsbad, CA, USA) and retrotranscribed into cDNA in two steps by SuperScript III First-Strand Synthesis System (Invitrogen, Carlsbad, CA, USA) according to the manufacturer’s instructions. Cell fractionation was carried out using Ambion PARIS kit according to manufacturer’s protocol. Nuclear and cytoplasmic RNA was extracted with Qiazol reagent and the miRNeasy spin columns (Qiagen, Hilden, Germany) according to the manufacturer’s specifications. RNase R treatment was performed as follows: 2 µg of total RNA was treated with 2u RNase R/µg (MRNA092, Epicentre Biotechnologies–Madison, WI, USA) for 15 min at 37 °C and purified by phenol chloroform extraction. Primers used for the PCR amplification of specific genes are shown in Table 1. For RT-PCR amplification specific primers used for circSMOX, Myogenin, ATP Synthase Peripheral Stalk Subunit OSC (ATP50) genes are listed in Table 1. PCR reactions were performed in a 50 μL reaction volume using DreamTaq DNA Polymerase (Thermo Fisher Scientific, Waltham, MA, USA) Reaction Kit in accordance with the manufacturer’s protocol. Amplification and digested products were analyzed by 1 and 2.5%. agarose gel electrophoresis according to the expected fragment molecular weight. qRT-PCR analyses were carried out by SYBR-Green method and corresponding specific primers are listed in Table 1. Reactions were performed in AriaMx Real-Time PCR System (Agilent Technologies) using the following program: 40 cycles of 95 °C for 2 min, 95 °C for 5 s, 60 °C for 30 s. The mRNA for the constitutive glyceraldehyde-3-phosphate dehydrogenase (GAPDH) was examined as the reference transcript. The data are calculated relative to the internal housekeeping gene according to the second derivative test [delta–delta Ct (2-ΔΔCT)] method. The relative levels of SMOX gene splice variants (µSMO and βSMO) were measured by qRT-PCR using a set of specific primers listed in Table 1 and based on the nucleotide sequence of the alternative splicing products described by Cervelli et al. [6]. 

### 2.7. Western Blot

Aliquots from C2C12 cells extracted samples containing 20–40 μg of proteins were analyzed by 4–15% SDS–polyacrylamide gel electrophoresis (PAGE) under reduced conditions. After electrophoresis, the proteins were transferred onto 0.2 µm Nitrocellulose blotting membranes Protran (Amersham Biosciences, GE Healthcare Europe GmbH, Glattbrugg, Switzerland). Nonspecific binding of proteins was blocked with 5% non-fat milk in Tris-buffer (TBST) containing 0.1% Tween 20 for 1 h at room temperature (RT). The membranes were subsequently incubated overnight with the following antibodies: rabbit polyclonal anti-SMOX (1:1000) (SAB1101510, Merk-Millipore Corporation, Darmstadt, Germany), rabbit polyclonal anti-MYH (1:1000) (sc-20641, Santa Cruz Biotechnology, Santa Cruz, CA, USA), rabbit monoclonal anti-Fbx32 (1:1000) (ab168372, Abcam plc, Cambridge Biomedical Campus, Cambridge, CB2 0AX, UK), mouse monoclonal anti-GAPDH (1:4000) (MAB374, Merk-Millipore Corporation, Darmstadt, Germany). Membranes were washed in TBST three times for 10 min and one time in TBS for 10 min. The chemiluminescent signals were detected using Clarity Western ECL Substrate Detection Reagent (Bio-Rad Laboratories, Inc., Hercules, CA, USA). Immunoblots were imaged using a ChemiDoc (Bio-Rad Laboratories, Inc., Hercules, CA, USA) and quantified using Image Lab software 5.2.1 (Bio-Rad Laboratories, Inc., Hercules, CA, USA). Protein determination was carried out according to Bradford method using bovine serum albumin as standard (Sigma-Aldrich, St. Louis, MO, USA).

### 2.8. Statistical Analysis

We used Prism software (GraphPad Software, San Diego, CA, USA) to check statistical significance. The results are presented as means ± SD of three independent experiments, each performed in triplicate. For the nuclear/cytoplasmic fractionation the results are presented as means ± SE of four independent experiments. Statistical evaluation was conducted between two groups by the Student’s *t*-test, and values significantly different from the relative control are indicated with an asterisk when *p* ˂ 0.05 and with two asterisks when *p* ˂ 0.01. Statistical analysis of experiments containing more than two-group comparison was performed by the one-way analysis of variance (ANOVA), followed by Bonferroni’s test. Values significantly different from the relative control are indicated with an asterisk when *p* ˂ 0.05, with two asterisks when *p* ˂ 0.01, with three asterisks when *p* < 0.001 and with four asterisks when *p* < 0.0001.

## 3. Results 

### 3.1. Evaluation of the Existence of a Circular RNA for SMOX 

**Figure 1:**
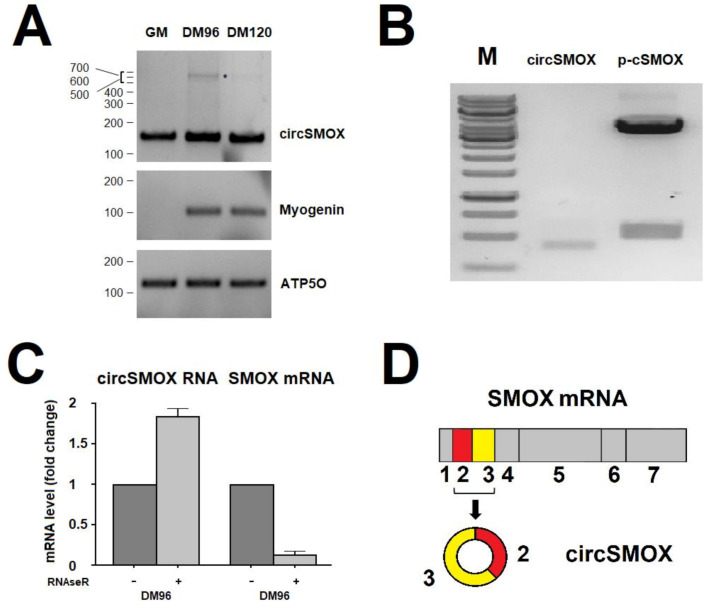
Evaluation of the existence of a circular RNA for SMOX. (**A**). RT-PCR analysis on murine C2C12 myoblasts (GM) and myotubes (DM) at different time points (DM 96 and DM 120) using primers circSMOX1-circSMOX 2 to amplify the circSMOX back splicing junction. The expected molecular weight (153 bp) of the PCR amplified product is visible in all lanes. The DM96 sample shows an additional band (asterisk) of 614 bp estimated size probably corresponding to a concatenamer. The expression of Myogenin transcript was used as control of differentiation while the ATP50 expression was used as loading control. The primers used for the RT-PCR are listed in Table 1. (**B**). Digestion analysis with *HindIII* and *XhoI* of the cloned product in pCR2.1 (p-cSMOX) after RT-PCR amplification (circSMOX) using primers circSMOX3 and circSMOX4. The circSMOX PCR product is 434 bp long and the digested product is 531 bp. Marker (M) peqGOLD 1kb DNA Ladder (VWR international, LLC) was used to calculate sample molecular weights. (**C**). Graph showing the levels of circular and and linear SMOX RNA measured by qRT-PCR on total RNA from murine myotubes (DM96) treated or not with RNAseR. (**D**). Schematic representation of SMOX mRNA and of the circSMOX arising from the circularization of the second and third exons.

Two independent studies identified a circRNA molecule arising from the second and third exon of SMOX primary transcript and showed its modulation in murine penumbral cortex 6 h after transient middle cerebral artery occlusion (MCAO; circ_000595) [21] and during muscle atrophy induced by denervation (circ-0001068) [22]. In order to study the expression of this circRNA, renamed circSMOX in this study, during in vitro muscle differentiation, the specific back splicing junction was amplified by RT-PCR using divergent primers circSMOX1-circSMOX 2 (Table 1) in murine C2C12 myoblasts (GM) and myotubes (DM) at different time points (DM 96 and DM 120) (Figure 1A). Electrophoretic analysis shows in all lanes a single band of about 150 bp corresponding to the expected size of 153 bp; an additional band probably corresponding to a concatenamer with an estimated size of 614 bp is visible in the DM96 sample. This circular amplified product is possibly generated by rolling circle retro-transcription as generally occurring on circular RNA substrates [42]. The expression of circSMOX paralleled the Myogenin expression an early marker of muscle differentiation. (Figure 1A). The amplified product (circSMOX) of the molecular weight of 434 bp, obtained by RT-PCR using the divergent primers circSMOX3- circSMOX4 (Table 1), from differentiated cells (DM 96) was cloned in pCR2.1 (p-cSMOX). The plasmid insert was then sequenced to confirm the junction sites of exons 2 and 3 (Figure 1B). Digestion analysis of p-cSMOX shows the plasmid insert with a molecular size of 531 bp. To rule out a trans-splicing reaction mechanism responsible for producing a linear RNA transcript consisting of a fusion of exons 3 and 2, RNA from murine myotubes was digested with the exoribonuclease RNAseR. As shown in Figure 1C, circSMOX RNA was resistant to RNAseR treatment; by contrast, the linear counterpart was efficiently degraded. Based on RT-PCR analysis, sequencing and RNAseR treatment a circular RNA for SMOX formed by the second and third exons was demonstrated being expressed in murine myotubes and depicted in Figure 1D.

### 3.2. RNA Transcript Levels during C2C12 Cell Differentiation 

**Figure 2:**
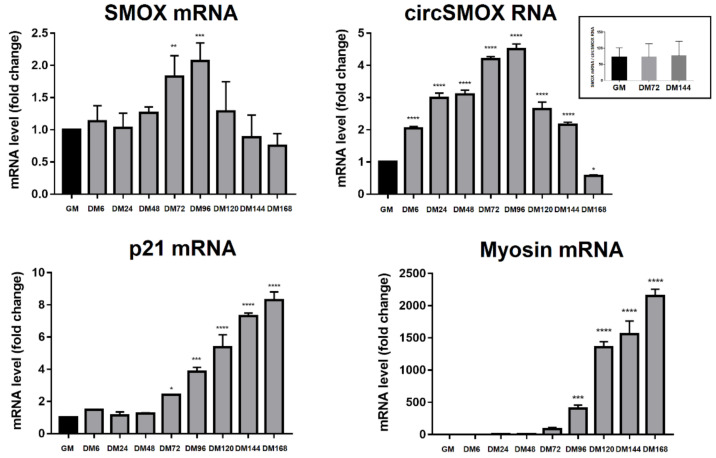
RNA transcript expression during C2C12 cell differentiation. qRT-PCR of linear SMOX, circSMOX, p21 and myosin RNAs amplified from C2C12 myoblasts (GM) and myotubes (DM) at different time points (DM 6–168). Data are calculated relative to the internal housekeeping gene (GAPDH) and are expressed as the mean fold change compared with GM control. Each value represents the mean ± SD of three independent experiments, each performed in triplicate. One-way ANOVA, followed by Bonferroni’s test, were used to determine. significant differences * *p* < 0.05, ** *p* < 0.01, *** *p* < 0.001, **** *p* < 0.0001 compared to GM control. In the insert, the ratio between linear and circular SMOX RNA expression is indicated.

The expression of linear SMOX, circSMOX RNA, p21 and myosin RNA transcripts have been analyzedanalyzed by quantitative RT-PCR (qRT-PCR) at different time points of C2C12 cell differentiation. As shown in Figure 2 the transcription level of the linear SMOX mRNA displays a bell-shaped curve during myogenesis. Its level increases during differentiation, showing the highest peak (about 2-fold increase compared to GM) of expression after 96 h (DM96) of differentiation stimuli, then it decreases during late differentiation. Interestingly, the expression curve of the circSMOX RNA parallels the linear counterpart, presenting a similar profile reaching the highest level of expression (about 4.5 fold increase compared to GM) after 96 h (DM96) of differentiation thus suggesting a transcriptional activation of the locus. Conversely, the p21mRNA displays a different expression curve, starting an increase at 72 h and then continuing to rise during differentiation. The myosin transcript level has been examined as a control of the C2C12 differentiation. As expected, myosin rapidly increases after 72 h post differentiation stimuli. 

### 3.3. SMOX Protein Level during C2C12 Cell Differentiation 

**Figure 3:**
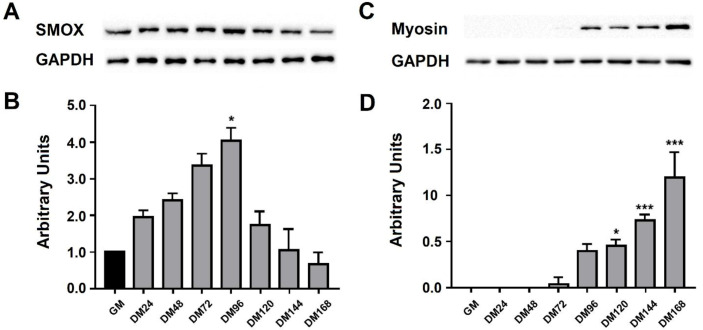
SMOX protein expression profile during C2C12 cell differentiation. Western blot analysis of SMOX (**A**) and Myosin (**C**) proteins on murine C2C12 myoblasts (GM) and myotubes (DM) at different time points (DM 24–168). Data are calculated relative to the internal housekeeping GAPDH gene, used as a loading control. The quantification of total levels of SMOX (**B**) and Myosin (**D**) were determined by densitometric analyses and expressed as arbitrary units. The values are presented as mean ± SD from three independent experiments, each performed in triplicate. One-way ANOVA, followed by Bonferroni’s test, were used to determine significant differences * *p* < 0.05, *** *p* < 0.001 compared to GM control.

The expression profile of SMOX protein has been analyzed by western blot at different time points during C2C12 cell differentiation. According to linear SMOX mRNA levels, the SMOX protein also exhibits a bell-shaped curve during myogenesis reaching the maximum level (about 4 fold increase compared to GM) at 96 h (DM96), as shown in Figure 3A,B. C2C12 cell differentiation was confirmed by myosin protein expression (Figure 3C,D).

### 3.4. Linear and Circular SMOX RNAs Expression in Atrophic C2C12 Cells

**Figure 4:**
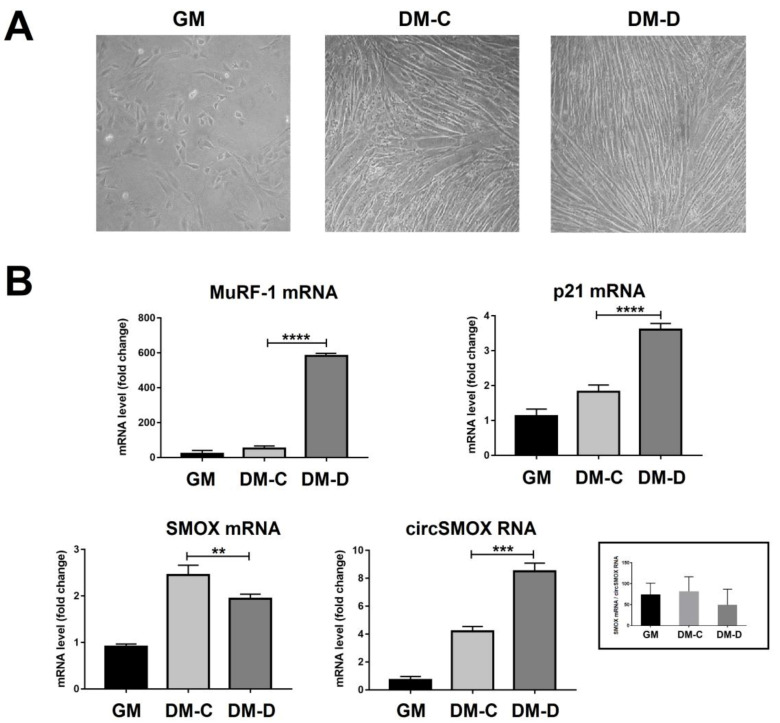
Linear and circular SMOX RNAs expression in atrophic C2C12 cells. (**A**). Microscopic analysis of C2C12 cells cultured on growth medium for 48 h (GM, myoblasts) were differentiated for 120 h (DM-C, untreated myotubes) or differentiated for 96 h and then treated with DEXA 100 µM for 24 h to induce atrophic condition (DM-D, treated myotubes). (**B**). qRT-PCR of MuRF-1 and p21 mRNAs (B) and of linear and circular SMOX RNAs (D) from GM, DM-C and DM-D. Data are calculated relative to the internal housekeeping gene (GAPDH) and are expressed as the mean fold change compared with GM control. Each value represents the mean ± SD of three independent experiments, each performed in triplicate. One-way ANOVA, followed by Bonferroni’s test, were used to determine significant differences ** *p* < 0.01, *** *p* < 0.001, **** *p* < 0.0001 compared to GM control.

**Figure 5:**
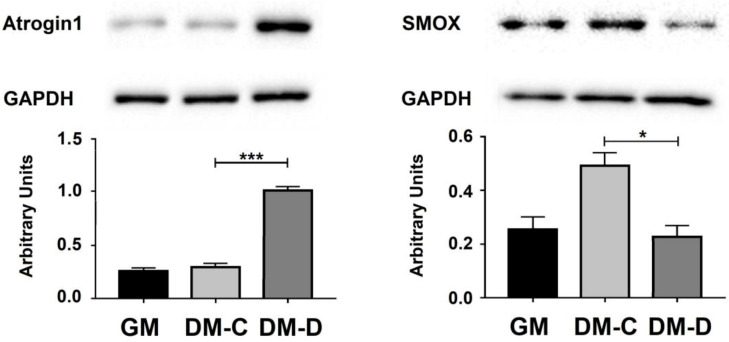
SMOX and Atrogin1 proteins expression profile in atrophic C2C12 cells. Western blot analyses of Atrogin1 (C) and SMOX (E) proteins on murine C2C12 GM, DM-C and DM-D. Data are calculated relative to the internal housekeeping GAPDH gene, used as a loading control. The quantification of total levels of Atrogin1 and SMOX proteins were determined by densitometric analyses and expressed as arbitrary units. The values are presented as mean ± SD from three independent experiments, each performed in triplicate. One-way ANOVA, followed by Bonferroni’s test, were used to determine significant differences * *p* < 0.05, *** *p* < 0.001 compared to GM control.

In order to induce an atrophic condition, C2C12 cells, after 96 h of differentiation, were treated with DEXA 100 µM (DM-D) or vehicle (DM-C) for 24 h (Figure 4A). The atrophy process was visually checked by microscopic analysis (Figure 4A) and confirmed by MuRF-1 and Atrogin1 expression (Figure 4B and Figure 5). Under atrophic condition (DM-D) the expression level of SMOX mRNA decreases (about 20% compared to DM-C); while the mRNA level of p21 increases (about 2-fold increase compared to DM-C) (Figure 4B). According to RNA expression, SMOX protein level was affected by DEXA treatment (DM-D) showing a relevant decrease (50%) compared to DM-C (Figure 5). Notably, during atrophic state, contrary to the expression of the linear form, the circSMOX RNA shows a relevant increase (about 2-fold increase compared to DM-C) (Figure 4B). 

### 3.5. SMOX RNA Transcript Splicing Variant Levels in Atrophic C2C12 Cells

**Figure 6:**
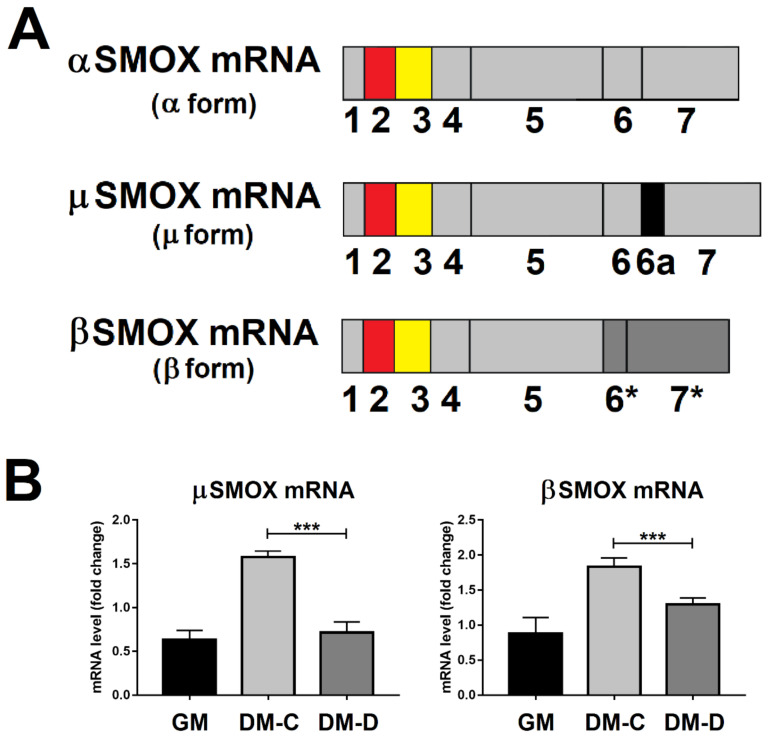
SMOX RNA transcript splicing variant levels in atrophic C2C12 cells. (**A**) Schematic representation of α, µ and β SMOX mRNA splicing variants. The second and third exons composing circSMOX RNA are highlighted in red and yellow respectively, common exons are in light grey, extra-exon present in the µ SMOX mRNA splicing variant, in light grey, extra-exon 6a present in the µ SMOX mRNA splicing variant, in dark grey, out of frame exons present in the β SMOX mRNA splicing variant. (**B**) qRT-PCR of µ and β SMOX mRNA splicing variants from GM, DM-C and DM-D. Data are calculated relative to the internal housekeeping gene (GAPDH) and are expressed as the mean fold change compared with GM control. Each value represents the mean ± SD of three independent experiments, each performed in triplicate. One-way ANOVA, followed by Bonferroni’s test, were used to determine significant differences *** *p* < 0.001 compared to GM control.

During the SMOX mRNA maturation many alternative processes can occur producing different transcript, among these, the major SMOX mRNA (α form), and the minor forms µ and β SMOX mRNA (Figure 6A), as observed in mouse brain [6] and C2C12 cells [3]. In order to examine whether the expression profile of these minor SMOX mRNA variants follows the same processing pathway of the major α form or eventually the expression profile of the circSMOX RNA during atrophy, qRT-PCR analyses have been performed. As shown in Figure 6B, the transcript level of these SMOX mRNA variants exhibits the same profile of the α SMOX form, decreasing after atrophic stimuli induced by DEXA.

### 3.6. Cellular Localization of Circular SMOX RNA in Atrophic C2C12 Cells

**Figure 7:**
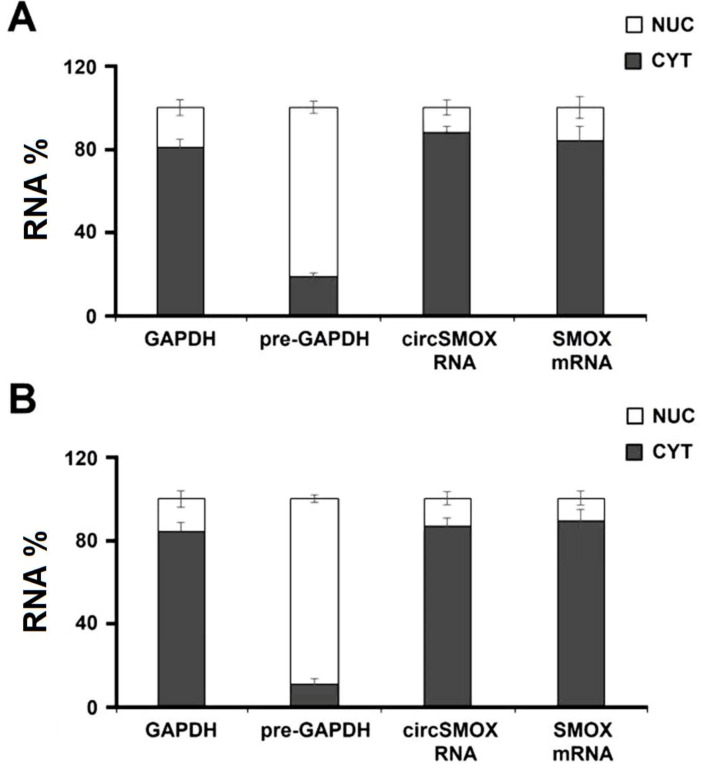
Cellular localization of circular SMOX RNA in atrophic C2C12 cells. The nuclear (NUC) and cytoplasmic (CYT) localization of linear and circular SMOX transcripts analyzed by qRT-PCR in murine myotubes untreated (**A**) and treated with DEXA (**B**). As control of the quality of the fractionation protocol, the precursor of GAPDH (pre-GAPDH) and the GAPDH transcripts have been used. RNA measurements were performed four independent experiments, each performed in triplicate. Bars show means and SE.

The sub-cellular localization of circSMOX RNA has been analyzed by qRT-PCR in murine myotubes. Notably, circSMOX resulted almost confined in the cytoplasm (Figure 7A) and this localization does not vary upon DEXA treatment (Figure 7B).

### 3.7. Linear and Circular SMOX RNA Expression in Two Mouse Models of Amyotrophic Lateral Sclerosis

**Figure 8:**
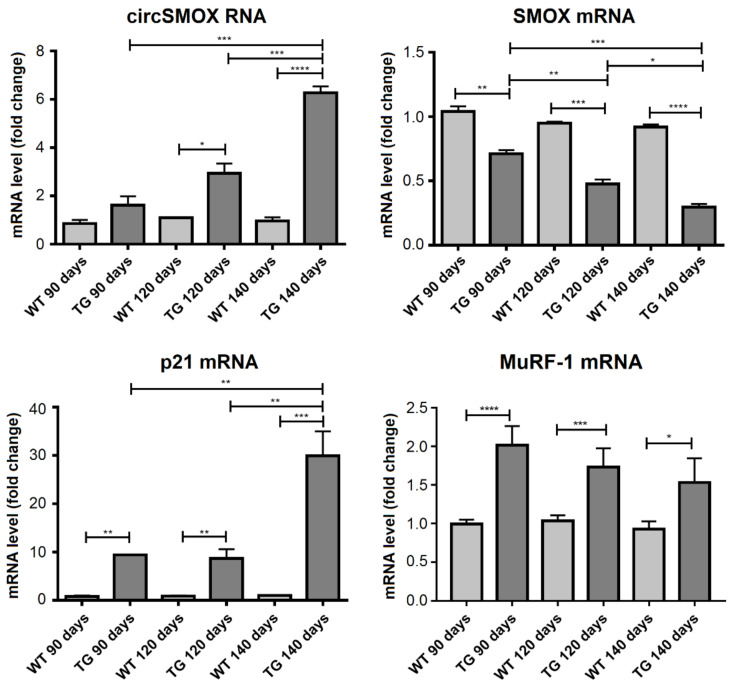
Linear and circular SMOX RNA expression in *SOD1^G93A^* mice. qRT-PCR of linear SMOX, circSMOX RNA, p21 and MuRF-1 RNA transcripts amplified from gastrocnemius of control (WT) and *SOD1^G93A^* mice (TG) at different stages of the disease; pre-symptomatic (90 days old), symptomatic (120 days old) and at the end stage for these animals (140 days old). Data are calculated relative to the internal housekeeping gene (GAPDH) and are expressed as the mean fold change compared with 90 days old WT controls. Each value represents the mean ± SD of three independent experiments. One-way ANOVA, followed by Bonferroni’s test, were used to determine significant differences * *p* < 0.05, ** *p* < 0.01, *** *p* < 0.001, **** *p* < 0.0001 compared to control mice.

**Figure 9:**
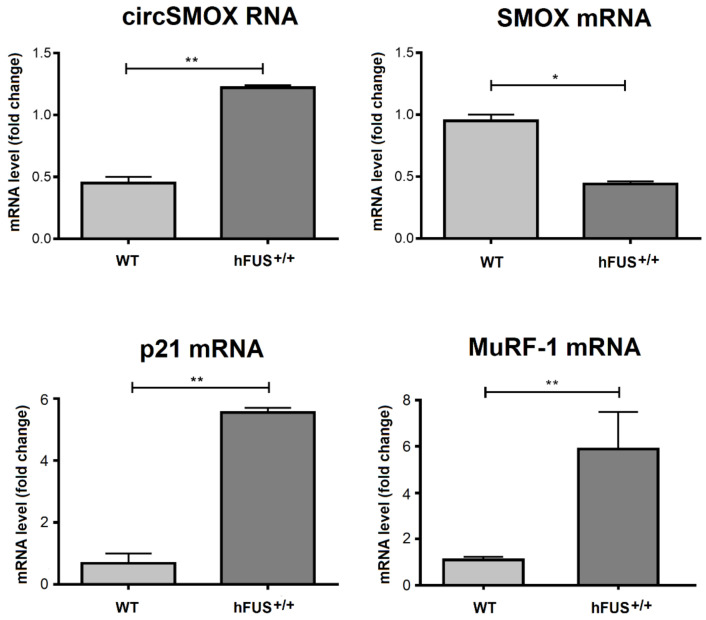
Linear and circular SMOX RNA expression in *hFUS^+/+^*mice. qRT-PCR of linear SMOX, circSMOX RNA, p21 and MuRF-1 RNA transcripts amplified from gastrocnemius of symptomatic 38 days old *hFUS^+/+^* mice (TG) and their control littermates (WT). Data are calculated relative to the internal housekeeping gene (GAPDH) and are expressed as the mean fold change compared with WT controls. Each value represents the mean ± SD of three independent experiments. Student’s t test was used to determine significant differences * *p* < 0.05, ** *p* < 0.01 compared to control mice.

The expression levels of the linear and circular SMOX transcripts have been examined in the two ALS mouse genetic systems *SOD1^G93A^* and *hFUS^+/+^.* In *SOD1^G93A^* mice, the levels of different transcripts were analyzed in the gastrocnemius at different stages of the disease; pre-symptomatic stage (90 days old), symptomatic stage (120 days old) and at the end stage for these animals (140 days old). As shown in Figure 8, SMOX mRNA level is significantly reduced in *SOD1^G93A^* mice compared to control ones in all stages analyzed and this decrease is more evident with the progression of the disease. By contrast, an increase of p21 mRNA level can be observed at 140 days old *SOD1^G93A^* mice, while the 90 and120 days old mice show a higher p21 transcript level compared to wild type control mice. With respect to circSMOX, in SOD1*^G93A^* mice compared to control ones at 120 days and at 140 days, a significant increase was observed (about nine times and 30 times respectively) indicating that the up-regulation of circSMOX parallels the progression of the disease. The transcript level of SMOX and p21 transcripts were also analyzed in the gastrocnemius of symptomatic 38 days old *hFUS^+/+^* mice. Also, in this ALS model a decrease of linear SMOX mRNA and an increase of both circSMOX RNA and p21mRNA were observed. MuRF-1 mRNA expression analysis confirmed to the progression of the disease in both ALS models (Figure 8 and Figure 9).

## 4. Discussion

Skeletal muscle is the most abundant tissue in the human body and its mass changes according to physiological or pathological conditions [2]. Maintaining muscle health and function is an important protection against chronic diseases and investigations into the mechanism of muscle differentiation are critical to design effective therapies for chronic disorders, such as atrophy and related pathologies. In this context, in the present study we have validated the existence of circSMOX RNA during myoblasts differentiation as well as in in vitro and in vivo atrophy conditions and extended the knowledge about the relationship between linear SMOX and p21 mRNAs in muscle physiopathology. These data, together with those from the literature [3,7,8] suggest that SMOX might be an anti-atrophy factor essential for the maintenance of skeletal muscle mass. It is noteworthy that SMOX expression is strongly repressed by p21, a protein that is highly induced by conditions inducing muscle atrophy but also expressed in time dependent way during differentiation [2,43]. Notably, the inverse relationship between these two proteins observed in muscle atrophy may be extended also to the process of differentiation even though possibly with a different significance. SMOX is an enzyme involved in animal cell polyamines homeostasis, selectively active on spermine and producing hydrogen peroxide (H_2_O_2_), spermidine (Spd), and the 3-aminopropanal. The high expression of SMOX in muscle tissue [6] can be related to hydrogen peroxide production, since this is a signaling molecule that controls the fate of mononuclear muscle cells [44,45,46,47]. We can speculate that at certain point of differentiation SMOX expression is important for the progression in myogenesis and when the H_2_O_2_ production is no more required, the increase in p21 turn off its expression. However, the anti-atrophic role of SMOX could be also related to the other SMOX reaction product Spd, which in turn can act on different autophagic pathways [2]. In particular, Spd can attenuate age-related skeletal muscle atrophy and diseases, through the regulation of autophagy via AMP-activated protein kinase (AMPK)/Protein Kinase B (AKT)/E1A binding protein p300 (EP300)-FOXO signal pathways (Cervelli et al., 2018 [2]).

Numerous evidences suggest that circRNAs, a novel class of covalently closed circular non-coding RNAs, with no 5′ caps and 3′ poly(A) tails, formed by back-splicing reaction, are involved in skeletal muscle pathophysiology [14,18,19,48,49]. 

However, the exact function of circRNAs in skeletal muscle growth and development is still unclear; therefore, the knowledge of their pattern of expression may be of great help to better comprehend their role and in such way to refine our understanding of myogenesis and myopathies.

Recently, in two high throughput studies on mice regarding muscle atrophy and transient focal ischemia [21,22], the existence of a back splicing junction potentially corresponding to a circRNA derived from the exons 2 and 3 of SMOX primary transcript has been detected though computational analyses, and here named circSMOX. In both cases, this putative circRNA resulted up-regulated in two different tissues, in particular in brain at six hours of perfusion after MCAO [21] and in the gastrocnemius after sciatic denervation [22]. 

In the present work, the existence of circSMOX has been validated by PCR using divergent oligonucleotides and the cloning and sequencing of this PCR product demonstrated that it was indeed composed by the fusion of exon 2 and 3 of SMOX transcript. Moreover, the circularity of circSMOX was verified by RNAseR treatment. Generally, circRNAs are expressed at low levels compared to linear mRNA counterparts [15,16,50,51,52], this is the case of circSMOX during skeletal muscle differentiation which is about 70 times less abundant than linear form; although showing the same expression profile. In line with the results of Weng and co-worker [22], we also observed an upregulation of circSmox when an atrophic state is induced in vitro. Notably, in atrophic cells circSmox expression showed an opposite trend compared to the linear form suggesting that in this condition the back splicing is somehow preferred respect to the canonical process. Moreover, during focal ischemia in mouse brain circSMOX resulted upregulated at 6 h of perfusion after MCAO and then drops to normal levels [21] while its linear counterpart increases until 24 h [53], thus reinforcing the idea that the production of the two RNA products might be tightly regulated under particular conditions. The possible alternative choice between linear and circular splicing has been already described for other circRNAs allowing them to be independently expressed respect their corresponding linear transcripts [16,52,54,55,56,57]. 

It is noteworthy that skeletal muscle is one of the tissues with the highest number of differentially expressed genes by alternative splicing, and abnormal intron removal has been largely implicated in the pathophysiology of muscle disease [58]. Our previous studies demonstrated that the linear form of SMOX goes through alternative splicing during C2C12 cells and mouse skeletal muscle [3,6]. To verify the alternative splicing in atrophic conditions, we analyzed SMOX gene splicing variants (µ and βSMOX mRNAs) by qRT-PCR. The analysis showed a decreased level after DEXA treatment similar to the major form of SMOX (αSMOX mRNA), indicating that splicing factors involved in the linear SMOX mRNA isoforms production are regulated in concert, opposed to the ones responsible for circSMOX RNA generation. 

As it has been observed in other instances, we found that the SMOX protein decreases following the trend of SMOX RNA. It is worth mentioning that such reduction could lead to a decrease in Spd, the other product of SMOX activity. Spd is of great interest for the prevention or treatment of muscle diseases and a decrease of spermidine cellular concentration is involved in skeletal muscle atrophy [59,60]. Hence, it is possible that a minor production of Spd can be an important contributor to the development of atrophy. 

One of the relevant findings in this study is the observation that linear and circular SMOX RNA are regulated differently during the atrophic response. Whereas linear SMOX is significantly reduced, circSMOX expression is increased by atrophy. In order to get insight into the function of circSMOX, we explored its intracellular localization; cellular distribution of circSMOX displays a prevalent cytoplasmic localization both in differentiated and atrophic C2C12 cells (Figure 7). It has been shown that in mouse and human muscle cells/tissues circular RNAs are almost exclusively located in the cytoplasm, according to their role of being sponges for miRNAs regulating their levels by competitive binding or to their coding capacity [14]. Examples of circRNAs acting as sponges for miRNAs and involved in the regulation of myogenesis are circLMO7 and circFGFR4. CircLMO7 serves as a sponge for miR-378a-3p and decreases the expression of MyoD and myogenin, inhibiting myoblast differentiation [49,61]; while circFGFR4 interacts with miR-107, inducing cell apoptosis and promoting myoblasts differentiation [49,62]. Our observations suggest that circSMOX may play a role during muscle atrophy in the cytoplasm, therefore, we can envisage that in this compartment it might act as sponge for miRNAs thus regulating the expression of atrophy related mRNA targets. Moreover, circZNF09 has been demonstrated to control myoblast proliferation in mouse and human and to hold the capacity to be translated [18]. Our observations suggest a potential regulatory role of circSMOX in the cytoplasm during muscle atrophy, therefore, we can envisage that in this compartment it might act as sponge for miRNAs, thus regulating the expression of atrophy related mRNA targets, or alternatevely it might produce a protein isoform, due to the presence of a putative ORF formed upon circularization. To extend our studies in vivo we have used two mouse models of ALS, since one of the principal causes of affliction in ALS is skeletal muscle atrophy, that leads to paralysis and death within a few years after onset [63,64]. So far, a pharmacological therapy for muscle atrophy does not exist because its genesis it is still not fully understood at the molecular level. The development of mouse models for ALS, has made possible to investigate the molecular basis of this disease in the pursuit of treating this disorder. The use of two ALS mouse models based on different genes mutation coding for SOD1, a protein involved in oxidative stress, and FUS, a protein associated with RNA processing, well represent the multifactorial pathogenesis of ALS and reinforce the obtained results.

In both our ALS mouse models, SOD1*^G93A^* and hFUS*^+/+^* an increased expression of circSMOX and a decreased expression of the linear SMOX mRNA were observed, confirming the opposite expression of these RNA molecules also in in vivo. Noteworthy, also in ALS mouse models p21 mRNA and the linear SMOX RNA show an opposite trend, suggesting a p21-mediated repression of SMOX as demonstrated in other atrophy mouse models [8]. In conclusion, this work has demonstrated that the expression of circSMOX RNA increases in atrophy. This circular RNA can be now investigated not only as an indicator of normal biological processes, but also as a potential diagnostic, prognostic, predictive and pharmacodynamic biomarker of pathogenic processes and pharmacologic response to therapeutic treatments. In particular, the detection of both SMOX mRNA and circSMOX RNA, which have opposite expression profiles in atrophic condition, can be utilized in diagnostic and therapeutic monitoring of ALS.

## Figures and Tables

**Table 1:** Primers used in this study.

Gene	PCR Method	Primers
ATP Synthase	RT-PCR	ATP5O Fwd: 5′-CAACCGCCCTGTACTCTGCT-3′ ATP5O Rev: 5′-GGATTCAGAACAGCCAGAGACAC-3′
circSMOX	qRT-PCR	circSMOX1 Fwd: 5′-GCCTGCTACCTTACCAACC-3′ circSMOX2 Rev: 5′-CACGACTGAGAGGGTCATC-3′
	RT-PCR	circSMOX3 Fwd: 5′-GACAGCCTCGTGTGGTGG-3′ circSMOX4 Rev: 5′-GGTCATCCGCACTGTCGC-3′
GAPDH	qRT-PCR	Fwd: 5′-GGTTGTCTCCTGCGACTTC-3′ Rev: 5′-GGTGGTCCAGGGTTTCTTAC-3′
MuRF-1	qRT-PCR	Fwd: 5′-GACAGTCGCATTTCAAAGCA-3′ Rev: 5′-AACGACCTCCAGACATGGAC-3′
Myogenin	RT-PCR	Fwd: 5′-TCCCAACCCAGGAGATCATT-3′ Rev: 5′-CATATCCTCCACCGTGATGC-3′
Myosin	qRT-PCR	Fwd: 5′-ATGATCTACACCTACTCGGG-3′ Rev: 5′-GTTCTCCCGATCTGTCAGC-3′
p21	qRT-PCR	p21 Fwd: 5′-GACCTGGGAGGGGACAAG-3′ p21 Rev: 5′-TGATAGAAATCTGTCAGGCTG-3′
SMOX	qRT-PCR	SMOX Fwd: 5′-ACTCCAAGAATGGCGTGGC-3′ SMOX Rev: 5′-CGACGCTGTTCTGACTCTC-3′
βSMOX	qRT-PCR	SMOX β Fwd: 5′-ACAGTTCACAGGTGGGCTC-3′ SMOX β Rev: 5′-CCTCGCGTGGCCAGAG-3′
µSMOX	qRT-PCR	SMOX µ Fwd: 5′-ACAGTTCACAGGGAACCCC-3′ SMOX µ Rev: 5′-GCTTCTATGCGCTGTCTTGG-3′

## Abbreviations

ALS	Amyotrophic lateral sclerosis
GM	C2C12 myoblasts
DM	C2C12 myotubes
circSMOX	circular SMOX
p21	cyclin-dependent kinase inhibitor p21
DEXA	dexamethasone
GAPDH	glyceraldehyde-3-phosphate dehydrogenase
ATP50	ATP Synthase Peripheral Stalk Subunit OSC
MuRF-1	muscle RING-finger protein-1
SM	skeletal muscle
Spd	spermidine
SMOX	spermine oxidase
